# Anaphylaxis Following Cefazolin Administration in Pregnancy Managed With Delayed Cesarean Delivery: A Case Report

**DOI:** 10.1155/cria/8831413

**Published:** 2026-07-27

**Authors:** Do Van Loi, Nguyen Thanh Hoi, Nguyen Thi Sim, Tran Quang Hiep, Phung Quang Thuy, Nguyen Minh Nghia, Dinh The Tien, Luu Thuy Hien, Tran Vuong The Vinh

**Affiliations:** ^1^ Anesthesiology Center, Phenikaa University Hospital, Hanoi, Vietnam; ^2^ Anesthesiology faculty, Phenikaa School of Medicine and Pharmacy, Phenikaa University, Hanoi, Vietnam, phenikaa-uni.edu; ^3^ Phenikaa School of Medicine and Pharmacy, Phenikaa University, Hanoi, Vietnam, phenikaa-uni.edu; ^4^ Phenikaa University Hospital, Hanoi, Vietnam; ^5^ Department of Surgery, Hai Phong University of Medicine and Pharmacy, Hai Phong, Vietnam, hpmu.edu.vn

**Keywords:** adrenaline, anaphylaxis, case report, cefazolin, cesarean delivery, pregnancy

## Abstract

Anaphylaxis during pregnancy is rare but potentially fatal. Limited evidence is available to guide decisions regarding delivery timing during maternal anaphylaxis. A 26‐year‐old woman at 38.5 weeks scheduled for elective cesarean delivery developed anaphylaxis immediately after prophylactic cefazolin infusion, presenting with hypotension, tachycardia, dyspnea, erythema, and facial edema. She was treated promptly with intramuscular and intravenous adrenaline, fluids, corticosteroids, oxygen, and left uterine displacement, achieving hemodynamic stabilization without biphasic reaction. Continuous fetal monitoring was reassuring, allowing cesarean section to be postponed. At 23 h, spontaneous labor commenced, and delivery was performed safely under spinal anesthesia following negative allergy testing. A healthy neonate was delivered, and both mother and infant recovered uneventfully. Anaphylaxis following cefazolin administration during pregnancy is extremely uncommon. This case suggests that, when fetal status remains reassuring, cesarean delivery may be postponed until maternal stabilization in selected patients.

## 1. Introduction

Anaphylaxis during pregnancy is a rare but potentially life‐threatening condition, with an estimated incidence of 1.5–3.8 cases per 100,000 pregnancies. Maternal cardiovascular collapse may compromise uteroplacental perfusion, resulting in fetal hypoxia, neurological injury, or even fetal death. Although prompt recognition and treatment are essential, the management of anaphylaxis in pregnant patients presents unique challenges because maternal resuscitation must be balanced against fetal well‐being [1].

Current recommendations generally advocate the same initial management principles used in nonpregnant patients, including the prompt administration of adrenaline and aggressive hemodynamic support. However, decisions regarding the timing of delivery remain complex and must be individualized according to maternal condition, gestational age, and fetal status. In particular, limited clinical evidence is available regarding cases in which maternal stabilization is achieved before delivery [2].

We report a case of clinically diagnosed cefazolin‐associated anaphylaxis in a woman at term pregnancy. Following successful maternal resuscitation and continuous fetal monitoring, cesarean delivery was postponed until maternal recovery, resulting in favorable maternal and neonatal outcomes. This case highlights the challenges involved in balancing maternal stabilization and delivery timing during obstetric anaphylaxis.

## 2. Case Presentation

### 2.1. Patient Background

Ethical approval was obtained from the Institutional Review Board of Phenikaa University Hospital (No. DT.050/25). A 26‐year‐old gravida 2 para 1 woman at 38.5 weeks of gestation was admitted in June 2025 for an elective repeat cesarean section. She had no history of drug or food allergies and no significant comorbidities. Her previous cesarean section had been uneventful.

### 2.2. Onset of Reaction

Prophylactic intravenous cefazolin was initiated in the operating theater. Within minutes, the patient developed generalized erythema, facial edema, dyspnea, tachycardia (121–125 bpm), and hypotension (85/50 mmHg). Oxygen saturation was 97%, and her body temperature was 36.5°C. Fetal heart rate was 128–131 bpm with normal variability.

### 2.3. Initial Management

Cefazolin infusion was immediately discontinued, and treatment was initiated with intramuscular adrenaline, supplemental oxygen, rapid intravenous fluid resuscitation, corticosteroids, and left uterine displacement. Because hypotension persisted despite the initial intervention, a second dose of intramuscular adrenaline was administered four minutes later, followed by continuous intravenous adrenaline infusion at 0.1 µg/kg/min. Maternal hemodynamic status improved rapidly, with blood pressure increasing from 85/50 to 100/50 mmHg and heart rate decreasing from 121–125 to 100–105 bpm within five minutes. Continuous fetal monitoring demonstrated reduced variability but no persistent bradycardia or other evidence of fetal compromise.

### 2.4. Observation and Recovery

The patient was transferred to the operating room for close maternal and fetal surveillance, with preparations in place for emergency cesarean delivery should maternal deterioration or fetal compromise occur. Continuous cardiotocography demonstrated transient minimal variability following maternal hypotension; however, fetal heart rate remained within the normal range without persistent bradycardia, recurrent decelerations, or other signs of ongoing fetal compromise.

Intravenous adrenaline infusion was maintained and titrated according to maternal hemodynamic response. Throughout treatment, maternal blood pressure remained stable without recurrent hypotension, and continuous fetal monitoring remained reassuring. After approximately nine hours, maternal symptoms had resolved completely, the adrenaline infusion was discontinued, and no biphasic reaction was observed.

### 2.5. Delivery and Outcomes

**FIGURE 1:**
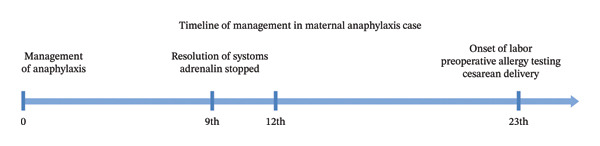
Timeline of management in maternal anaphylaxis case.

Following stabilization, serial multidisciplinary assessments involving obstetricians, anesthesiologists, allergists, and intensive care specialists confirmed sustained maternal recovery and reassuring fetal status. Given the absence of persistent maternal instability or fetal compromise, continued observation was considered appropriate and immediate cesarean delivery was deferred. Twenty‐three hours after the anaphylactic event, spontaneous labor commenced. After confirming maternal stability, cesarean delivery was performed under spinal anesthesia. A healthy female neonate weighing 3250 g was delivered with Apgar scores of 9 and 10 at one and five minutes, respectively. Both mother and infant had an uneventful postoperative recovery. The management timeline is summarized in Figure 1.

## 3. Discussion

This report describes successful management of maternal anaphylaxis and a favorable maternal and neonatal outcome following the postponement of cesarean delivery until after maternal stabilization.

Most episodes are reported intrapartum or during cesarean section, commonly linked to antibiotics, anesthetic agents, or latex exposure [1]. Previous reports of cefazolin‐induced anaphylaxis in pregnancy have described different management strategies and outcomes. Konno and Nagase reported a case occurring during labor in which maternal hypotension was accompanied by persistent fetal bradycardia, leading to emergency cesarean delivery and severe neonatal acidosis despite maternal recovery. In contrast, Daoud et al. described cefazolin‐induced anaphylaxis during the second trimester that was successfully treated with epinephrine and supportive care, allowing continuation of a fetoscopic procedure after stabilization. Our case differs from both reports because the patient was at term and scheduled for cesarean delivery, yet reassuring fetal status after maternal resuscitation allowed surgery to be postponed for 23 h until complete recovery. This experience suggests that, in selected cases with stable maternal hemodynamics and reassuring fetal monitoring, immediate delivery may not always be required [3, 4]. Although serum tryptase and allergen‐specific IgE testing may assist in confirming the diagnosis, they were not performed in this case. Therefore, the diagnosis was established clinically based on the immediate onset of symptoms following cefazolin administration, the characteristic manifestations of anaphylaxis, and the favorable response to standard treatment. Although pregnancy is often regarded as a relative contraindication for allergy testing, targeted testing may be justified in select situations where certain drugs are indispensable, such as local anesthetics [1].

Risk factors for anaphylaxis in pregnancy are not fully established. A history of allergy remains the most consistently reported risk factor. The differential diagnosis is broad, including amniotic fluid embolism, neuraxial hypotension, and pulmonary embolism, but the acute onset of symptoms during antibiotic infusion and the favorable response to adrenaline strongly supported the clinical diagnosis of anaphylaxis [5].

Adrenaline remains the cornerstone of treatment, despite concerns regarding uterine vasoconstriction and possible fetal hypoxia. Evidence consistently shows that delays in adrenaline administration are associated with poor outcomes [6]. In this case, the patient received repeated intramuscular doses followed by intravenous infusion, resulting in effective hemodynamic stabilization. This supports current recommendations that adrenaline should not be withheld in pregnancy and that aggressive resuscitation with fluids and positioning maneuvers are essential.

**FIGURE 2:**
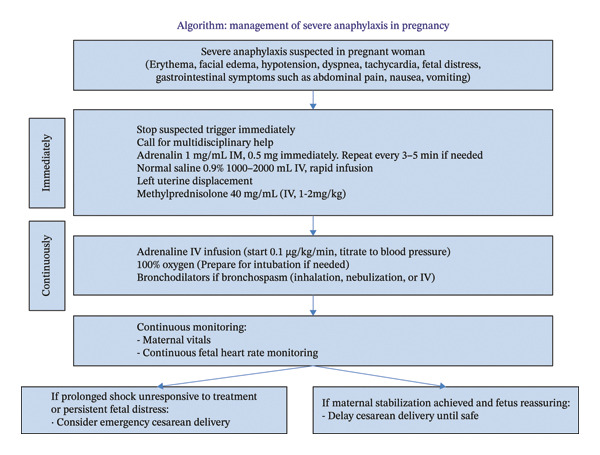
Algorithm: management of severe anaphylaxis in pregnancy.

A major clinical dilemma in obstetric anaphylaxis is determining the optimal timing of delivery. Emergency cesarean section is frequently performed in reported cases, particularly when fetal distress is present. However, anesthesia and surgery during ongoing maternal shock may expose both mother and fetus to additional risks due to unstable hemodynamics and potential exposure to further allergens. In the patient, continuous fetal monitoring demonstrated only transient minimal variability without persistent bradycardia or other signs of ongoing fetal compromise. This reassuring fetal status allowed maternal stabilization to be prioritized before proceeding to delivery. Importantly, the decision to postpone delivery was not based on a predefined protocol but on serial multidisciplinary assessments demonstrating sustained maternal recovery and reassuring fetal status. Following recovery, cesarean delivery was performed under spinal anesthesia with favorable maternal and neonatal outcomes. Figure 2 summarizes a practical approach to the management of severe anaphylaxis in pregnancy based on the present case and available literature. This case suggests that, in selected patients with successful maternal stabilization and reassuring fetal status, the postponement of cesarean delivery may be considered. However, this observation is derived from a single case and should be interpreted with caution.

This report had several limitations. First, it described a single patient and, therefore, cannot establish causal relationships or support broad clinical recommendations. Second, serum tryptase and allergen‐specific IgE testing were not performed, precluding definitive confirmation of cefazolin as the causative agent. Nevertheless, the close temporal relationship between cefazolin administration and symptom onset, together with the characteristic clinical manifestations and rapid response to standard treatment, strongly supported the clinical diagnosis of anaphylaxis. Finally, the favorable outcome observed in this case may not be generalizable to all pregnant patients experiencing anaphylaxis.

## 4. Conclusion

This case illustrates the importance of prompt recognition and treatment of anaphylaxis during pregnancy. Successful maternal stabilization combined with continuous fetal monitoring allowed individualized decision‐making regarding delivery timing. Further evidence is required before broader recommendations can be made.

## Author Contributions

Do Van Loi: conceptualization, methodology, writing–original draft, writing–review and editing, and supervision. Nguyen Thanh Hoi, Nguyen Thi Sim, Tran Quang Hiep, Phung Quang Thuy, Nguyen Minh Nghia, Dinh The Tien, Luu Thuy Hien, and Tran Vuong The Vinh: investigation, data curation, formal analysis, writing–original draft, and writing–review and editing.

## Funding

The authors have nothing to report.

## Disclosure

All authors have read and agreed to the published version of the manuscript.

## Ethics Statement

Ethical approval was obtained from the Institutional Review Board of Phenikaa University Hospital (Chairman: Prof. Do Quyet, No. DT.050/25).

## Consent

Informed consent was obtained from the patient.

## Conflicts of Interest

The authors declare no conflicts of interest.

## Data Availability

Corresponding authors take full responsibility for the data, analyses, and interpretation of the data and for providing accurate data availability policies.
